# Drug functional remapping: a new promise for tumor immunotherapy

**DOI:** 10.3389/fonc.2025.1519355

**Published:** 2025-03-14

**Authors:** Jiayi Dong, Ting Su, Jiexiong Wu, Yu Xiang, Minghan Song, Canfeng He, Lijuan Shao, Yubin Yang, Size Chen

**Affiliations:** ^1^ Department of Immuno-Oncology, The First Affiliated Hospital of Guangdong Pharmaceutical University, Guangdong Pharmaceutical University, Guangzhou, China; ^2^ Guangdong Provincial Engineering Research Center for Precision Medicine in Esophageal Cancer, Guangdong Pharmaceutical University, Guangzhou, China; ^3^ Key Laboratory of Monitoring Adverse Reactions Associated with Chimeric Antigen Receptor T-Cell Therapy, Guangdong Higher Education Institutions, Guangdong Pharmaceutical University, Guangzhou, China; ^4^ School of Clinical Medicine, Guangdong Pharmaceutical University, Guangzhou, China; ^5^ Traditional Chinese Medicine Department, West China Second University Hospital, Sichuan University, Chengdu, China; ^6^ Key Laboratory of Birth Defects and Related Diseases of Women and Children (Sichuan University), Ministry of Education, Chengdu, China

**Keywords:** old medicine new use, drug functional repositioning, tumor immunotherapy, immune sensitization, cancer

## Abstract

The research and development of new anti-cancer drugs face challenges such as high costs, lengthy development cycles, and limited data on side effects. In contrast, the clinical safety and side effects of traditional drugs have been well established through long-term use. The development or repurposing of traditional drugs with potential applications in cancer treatment offers an economical, feasible, and promising strategy for new drug development. This article reviews the novel applications of traditional drugs in tumor immunotherapy, discussing how they can enhance tumor treatment efficacy through functional repositioning, while also reducing development time and costs. Recent advancements in cancer immunotherapy have revolutionized treatment options, but resistance to ICIs remains a significant challenge. Drug repurposing has emerged as a promising strategy to identify novel agents that can enhance the efficacy of immunotherapies by overcoming ICI resistance. A study suggests that drug repositioning has the potential to modulate immune cell activity or alter the tumor microenvironment, thereby circumventing the resistance mechanisms associated with immune checkpoint blockade. This approach provides a rapid and cost-effective pathway for identifying therapeutic candidates that can be quickly transitioned into clinical trials. To improve the effectiveness of tumor immunotherapy, it is crucial to explore systematic methods for identifying repurposed drug candidates. Methods such as high-throughput screening, computational drug repositioning, and bioinformatic analysis have been employed to efficiently identify potential candidates for cancer treatment. Furthermore, leveraging databases related to immunotherapy and drug repurposing can provide valuable resources for drug discovery and facilitate the identification of promising compounds. It focuses on the latest advancements in the use of antidiabetic drugs, antihypertensive agents, weight-loss medications, antifungal agents, and antiviral drugs in tumor immunotherapy, examining their mechanisms of action, clinical application prospects, and associated challenges. In this context, our aim is to explore these strategies and highlight their potential for expanding the therapeutic options available for cancer immunotherapy, providing valuable references for cancer research and treatment.

## 1 Introduction

**Figure 1:**
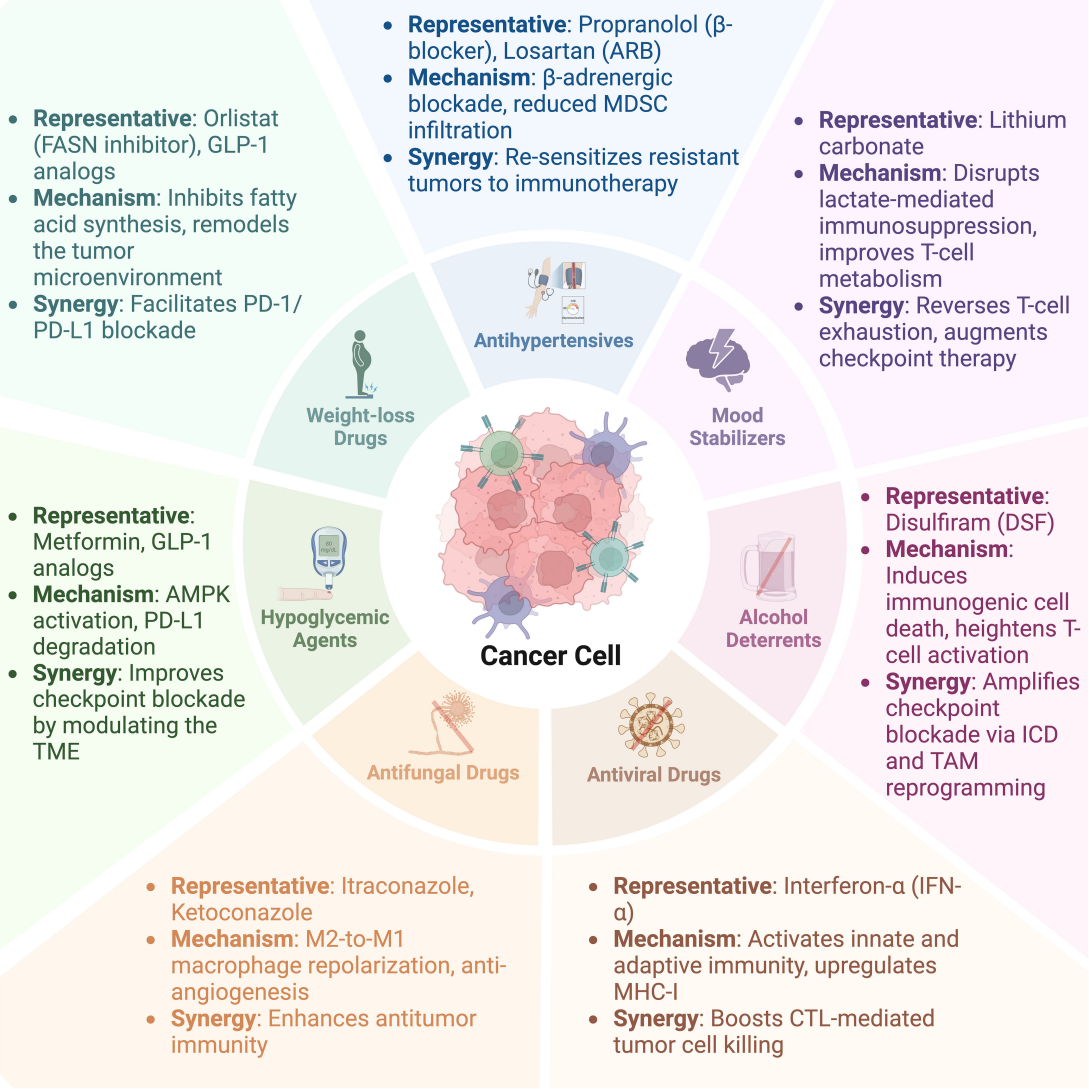
Schematic representation of repurposed conventional drugs in cancer immunotherapy. This figure illustrates seven categories of conventional medications—hypoglycemic agents, antihypertensives, weight-loss drugs, antifungal drugs, antiviral drugs, mood stabilizers, and alcohol deterrents—arranged around a central tumor cell. Each sector highlights representative agents, their principal mechanisms (e.g., metabolic reprogramming, induction of immunogenic cell death), and their capacity to enhance tumor immunity (such as augmenting checkpoint blockade). By modulating the tumor microenvironment and immune cell function, these “old drugs with new uses” show promise in strengthening antitumor immune responses and improving clinical treatment outcomes.

As tumor treatment methodologies continue to evolve, conventional approaches prove insufficient in addressing the complexities and variability of tumors. As a groundbreaking therapeutic strategy, tumor immunotherapy aims to disrupt the immune evasion mechanisms employed by tumor cells, reinitiate and sustain the tumor immune cycle, and restore the body’s normal anti-tumor immune response. Through various modalities—including monoclonal antibodies, immune checkpoint inhibitors, therapeutic antibodies, cancer vaccines, cell therapy, and small molecule inhibitors—immunotherapy can precisely target tumors and has the potential for personalized treatment, paving a new avenue for tumor management. In this context, the concept of repurposing old drugs has emerged. Scientists are able to efficiently identify drug candidates with repurposing potential by utilizing phenotypic screening, CRISPR/Cas9 functional screening, and virtual screening combined with machine learning. These methods rely on high-throughput experimental techniques and computational models, specifically focusing on discovering novel drugs that can reverse T-cell exhaustion, regulate tumor immune function, or ameliorate the immunosuppressive tumor microenvironment (1). In addition to their unique mechanisms of action, many traditional drugs have demonstrated significant efficacy in regulating the immune system, providing fresh perspectives for tumor treatment. For instance, recent studies have shown that metformin inhibits tumor cell growth and metastasis, with its mechanism linked to immune regulation. Similarly, antihypertensive agents have been found to enhance immune cell activity and promote anti-tumor immune responses, underscoring the potential of established drugs in tumor immunotherapy and offering valuable insights for further exploration of novel applications for these agents. This review aims to systematically summarize the new advancements of old drugs in tumor immunotherapy, analyze their potential mechanisms, and anticipate their broad applicability (**Figure 1**; **Table 1**). By uncovering the new value of old drugs, this research seeks to inject renewed vitality into tumor treatment and bring greater benefits to patients.

**Table 1:** The immunosensitizing effect of old drugs on solid tumors has been observed in recent studies.

Classes of Drugs	Research	The literature
Met	CRC: The immune microenvironment was reshaped to increase the proportion of CD8+ T cells and decrease the proportion of CD4+ T cells. Increased proportions of macrophages, pDCs, cDC1s, and mast cells and decreased proportions of neutrophils increased CD8+T cell toxicity.	(14)
	ESCC: It can enhance the regulation of T cell and macrophage population and affect the process of esophageal carcinogenesis.	(15)
	HNSCC: Patients who received Met before surgery had enhanced tumor infiltration of NK cells, which inhibited the CXCL1 pathway, while stimulating the STAT1 pathway within HNSCC NK cells, which enhanced NK cytotoxicity and prolonged overall survival when combined with chemoradiotherapy.	(19)
	MM: Combined with PD-1 antibody, CD8+T cell infiltration was synergistically induced and inhibited Mdscs-driven immunosuppression.	(20)
	PDAC: It can reduce the number and activity of tumor-friendly M2 macrophages and up-regulate the expression of FLT3, a key gene that affects the function of DC cells, thereby improving the immune status of TME.	(23)
Antihypertensive	BC: Reduce the elevation of peripheral Tregs caused by surgical stress.	(34)
	Fibrosarcoma, CRC: The anti-tumor effect was demonstrated by reducing tumor angiogenesis, promoting anti-tumor microenvironment, increasing T cell infiltration and reducing MDSC infiltration.	(35)
	MM: Combined ICI significantly increased the number of cytokines produced by CD8+T cells, shrank the tumor, and increased the expression of PD-1 and TIM-3, the markers of T-cell activation failure.	(36)
	Multiple immunocompetent tumor models: It can directly activate macrophages and promote the recruitment and activation of CD4+ and CD8+T lymphocytes in TME.	(37)
	PDAC: Significantly reduce the number of immunosuppressive Treg and FOXP3+ tumor cells, and increase the infiltration of antitumor CD8+ T cells.	(38)
	Cervical cancer: To inhibit tumor growth by regulating NFAT2 expression and enhancing tumor immune response to PD1ab.	(40)
Weight-loss Drugs	HCC: Inhibiting FABP1 activity can alleviate the progression of HCC, and the combination of FABP1 and PD-1 inhibitors has a significant effect.	(42)
Antifungal agents	Cervical cancer: Repolarize tumor-promoting M2 tumor-associated macrophages.	(49)
antiviral drug	MM: Combination with PD-1 blockade enhances efficacy, irrespective of a history of treatment with either IFN-α or ipilimumab.	(50, 51)
	HCC: The induction of tumor cells to secrete the chemokine CCL4 recruits cytotoxic CD8+ T cells, thereby enhancing therapeutic efficacy.	(52)
	CRC, Cervical cancer: It converts lactate into energy for CD8+ T cells, improves the activity of tumor-infiltrating CD8+ T cells, affects intracellular pH and epigenetic processes to regulate T cell immune responses.	(58)
	Liver cancer: It can promote the oxidation of lactic acid into mitochondria to restore and enhance the anti-tumor immune function of CD8+ T cells. Enhanced nitric oxide production and altered arginase activity significantly enhanced the functional activity of macrophages.	(59, 60)
DSF	CRC: DSF/Cu induces the expression of ICD signaling molecules and enhances the immune response.	(66)
	MM, CRC: DSF activates Lck-mediated TCR signaling to induce robust antitumor immunity.	(67)
	HCC: Inhibition of PARP1 by DSF/Cu with Cu2+ inhibited GSK3β activity, resulting in up-regulation of PD-L1 expression.	(69)
	Cervical cancer: DSF/Cu promotes the release of type I interferon and the maturation of DC, and in combination with DTX, it has a significant effect on metastatic breast cancer.	(71)
	NSCLC: Combined with anti-PD-L1, it regulates HIF-1 signaling pathway, eliminates drug resistance, and enhances cytotoxicity.	(72)
	PDAC: Combined chemotherapy and immunotherapy could significantly inhibit tumor growth by up-regulating the expression of IFNα and IFNβ.	(73)
	TNBC: It hypomethylated DNMT1-mediated IRF7 and enhanced PD-L1 expression.	(74)

## 2 Manuscript

### 2.1 Sensitizing effect of antidiabetic drugs on tumor immunotherapy

Traditional antidiabetic drugs, such as Metformin (Met), have shown remarkable potential in cancer treatment, exhibiting effects such as metabolic regulation, anti-cancer properties, and immune modulation. Metformin, a small molecule drug commonly used for treating type 2 diabetes, has demonstrated metabolic regulatory effects (2), tumor-suppressive properties, and immunomodulatory capabilities.

**Figure 2:**
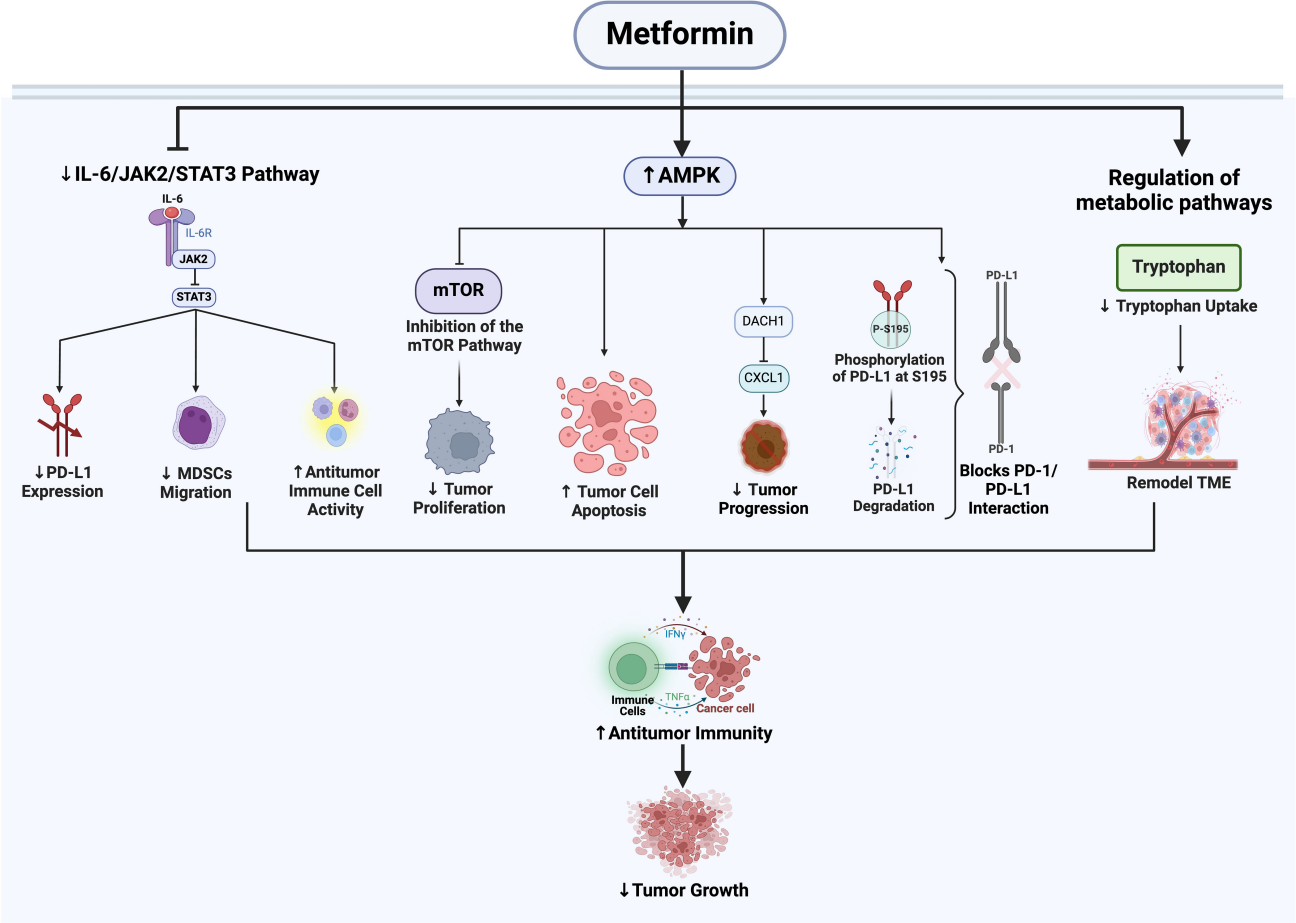
Diagram of the mechanisms by which metformin enhances tumor immunity. This figure delineates the action pathways of metformin in enhancing the tumor immune response through three primary mechanisms: activating AMP-activated protein kinase AMPK, inhibiting the IL-6/JAK2/STAT3 pathway, and regulating metabolic pathways.

Met exerts a direct anti-tumor effect by inhibiting cell proliferation and inducing apoptosis. It significantly inhibits proliferation and induces apoptosis in various tumor cell types through modulation of both AMPK-dependent and AMPK-independent pathways (3, 4). Furthermore, it reduces tumor incidence and enhances chemosensitivity (5). Enhanced sensitivity to radiotherapy and chemotherapy: Metformin increases the sensitivity of tumor cells to both radiotherapy and chemotherapy by inhibiting DNA damage repair, regulating the cell cycle, and enhancing cellular metabolism. Additionally, Metformin has multifaceted effects on both the innate and adaptive immune systems (6, 7). It can activate dendritic cells (DCs), promote the proliferation of CD4+ T cells (8), and sustain the activity of cytotoxic T lymphocytes (CTLs). Furthermore, it plays a crucial role in the anti-tumor immune response by regulating the differentiation and activity of peripheral regulatory T cells (Tregs) (9). By increasing the secretion of tumor necrosis factor (TNF-α) (10) and reducing the number of immunosuppressive cells such as myeloid-derived suppressor cells (MDSCs) (11), Metformin can reshape the tumor immune micro-environment and promote tumor cell death. Moreover, it effectively blocks the PD-1/PD-L1 axis (12) by activating AMPK to phosphorylate PD-L1 at serine 195, inducing abnormal glycosylation and degradation of PD-L1. Additionally, it enhances the efficacy of CTLA-4 inhibitors without increasing toxicity, regulates the immune system, and strengthens anti-tumor immune responses. Recent studies have demonstrated that Metformin can inhibit the secretion of pro-inflammatory cytokines by senescent T cells and reduce the expression of the senescence-associated secretory phenotype (SASP), thereby exerting an anti-aging effect and further enhancing the immune response (13) (**Figure 2**).

Through an in-depth exploration of the anti-tumor effects of Metformin and its immunomodulatory mechanisms, researchers have achieved significant findings. These studies confirm Metformin’s ability to inhibit tumor cell proliferation and induce apoptosis by modulating AMPK-dependent and AMPK-independent pathways, while elucidating its unique role in remodeling the tumor immune micro-environment, enhancing sensitivity to radiotherapy and chemotherapy, and regulating the immune system.

#### 2.1.1 Remodeling of tumor immune microenvironment and immune regulation

Research on Metformin in colorectal cancer (CRC) has shown that it can affect the metabolic pathways of tumor cells and reduce tryptophan uptake, thereby altering the composition of the tumor immune micro-environment (TME). Through single-cell transcriptome analysis, researchers observed an increased proportion of CD8+ T cells and a decreased proportion of CD4+ T cells in the colon tissues of APCmin/+ mice treated with Metformin, alongside an elevated presence of macrophages, plasmacytoid dendritic cells (pDCs), classical dendritic cells 1 (cDC1s), and mast cells. Conversely, the percentage of neutrophils was reduced (14). These alterations contribute to overcoming the immunosuppressive state associated with CRC and offer a novel strategy for immunotherapy.

In studies of esophageal squamous cell carcinoma (ESCC) and other tumor types, Metformin has significantly inhibited disease progression by improving the immunosuppressive tumor micro-environment and immune evasion mechanisms (15). Through various signaling pathways, such as the AMPK/DACH1/CXCL1 axis (16) and the IL-6/JAK2/STAT3 pathway (17), Metformin influences immune cell homeostasis. Combination therapy with PD-1 inhibitors can synergistically enhance anti-tumor immune responses while inhibiting PD-L1 expression. It reduces the migration of immunosuppressive cells, such as MDSCs, and increases the activity of anti-tumor immune cells, including CD8+ T cells and natural killer (NK) cells (18). These findings provide new targets and strategies for tumor immunotherapy.

#### 2.1.2 To improve the sensitivity of chemoradiotherapy and its clinical application

Metformin has also demonstrated a significant effect in enhancing sensitivity to chemoradiotherapy. By inhibiting DNA damage repair, regulating the cell cycle, and improving cellular metabolism, Metformin can increase the sensitivity of tumor cells to both radiotherapy and chemotherapy, thereby enhancing therapeutic efficacy. In a phase I trial involving head and neck squamous cell carcinoma (HNSCC), patients received 500 mg of metformin daily for 3 days, followed by 1000 mg daily until the day of surgery, for a minimum of 9 days. This regimen of metformin combined with chemoradiotherapy significantly prolonged overall survival and enhanced the activity of NK cells, CD4+ T cells, and CD8+ T cells (19). Additionally, significant progress has been made in the research of Metformin in malignant melanoma (MM) and pancreatic ductal adenocarcinoma (PDAC). In MM, the combination of Metformin and PD-1 antibodies has shown notable antitumor synergistic effects, enhancing the antitumor activity of PD-1 blockade (20) and improving overall survival, although the sample size was relatively small (55 patients) (21). Furthermore, large-scale clinical trials have confirmed that Metformin does not diminish the efficacy of the PD-1 antibody pembrolizumab nor worsen the prognosis for BRAF-mutated MM (22). In PDAC, metformin enhanced the immune status of the TME by inhibiting the infiltration of M2-type tumor-associated macrophages (TAMs) and increasing the infiltration and functionality of immune-activated DCs. In this study, patients were further stratified based on their metformin usage. Those who had been taking metformin for at least 6 months at the time of randomization and adhered to the standard metformin dosage recommended by their treating physicians (ranging from 500 to 2000 mg daily) were classified as “metformin users.” Patients with no history of metformin use were defined as “non-metformin users” (23). Although Metformin did not significantly improve survival in patients with PDAC when used alone in certain clinical trials (24), its potential benefits for patients with early-stage pancreatic tumors warrant further investigation.

#### 2.1.3 Exploration of innovative drug delivery systems and new hypoglycemic drug

In addition to traditional delivery methods, Metformin has been utilized as a novel immune adjuvant to significantly reverse immunosuppression (25) and enhance anticancer immunity in conjunction with photodynamic therapy or chemotherapy. Innovative drug delivery systems, such as novel biomimetic “Gemini nanoimmunomodulators,” have been developed to target tumor cells, PD-L1 checkpoints, and TAMs in a spatiotemporal manner. The precise and safe delivery of Metformin to tumor sites enhances the efficacy of photoimmunotherapy (26). Furthermore, studies have indicated that new glucose-lowering agents, such as glucagon-like peptide-1 (GLP-1) analogues, can offer new strategies and methodologies for tumor immunotherapy by improving NK cell function among other mechanisms (27).

In summary, the research findings regarding Metformin’s anti-tumor effects and its immunomodulatory mechanisms are substantial, providing not only new strategies and methodologies for tumor treatment but also revitalizing the development of immunotherapy. The achievement of these results is a testament to the relentless efforts and exploratory spirit of researchers, offering valuable insights and guidance for future investigations in tumor immunotherapy.

### 2.2 Traditional antihypertensive drugs and their role in tumor immunotherapy

Traditional antihypertensive agents include angiotensin-converting enzyme inhibitors (ACEIs), angiotensin II receptor blockers (ARBs), β-receptor antagonists, calcium channel blockers (CCBs), diuretics, and α2 receptor agonists (28). Numerous studies in recent years have demonstrated that these agents may exert indirect or direct effects on the tumor immune micro-environment by altering tumor angiogenesis and blood supply, thereby influencing tumor growth and immune responses (29).

#### 2.2.1 β-receptor antagonists

β-receptor antagonists, exemplified by propranolol, have shown the capacity to inhibit tumor growth and metastasis across various tumor models (30, 31). Preclinical and clinical studies indicate that propranolol can modulate the functional activity of immune cells, restore the vitality of cytotoxic T cells, and reduce the population of MDSCs. Furthermore, propranolol has been shown to enhance the anti-tumor efficacy of ICIs, offering a novel strategy for tumor immunotherapy.

In the tumor micro-environment, both tumor cells and immune cells frequently express ADRB (32). Activation of ADRB can lead to an increase in MDSCs and enhance immunosuppressive functions in a mouse model of breast cancer; however, propranolol can inhibit ADRB signal transduction and reduce the population of MDSCs in the spleen and tumors of tumor-bearing mice (33). In a clinical trial, patients were randomly assigned to one of four groups: a control group, a propranolol group, a parecoxib group, and a combination group receiving both propranolol and parecoxib. Patients in the propranolol group were administered oral propranolol (20 mg, three times daily) from the day of surgery to postoperative day 3. In the parecoxib group, patients received intravenous parecoxib (40 mg once daily) from the day of surgery to postoperative day 2. In the combination group, patients received both propranolol (orally) and parecoxib (intravenously) according to the same schedules. Peripheral blood samples (10 mL) were collected from each patient at five time points: on the morning of surgery (preoperatively, Pre-OP), at the end of surgery (End-OP), and on postoperative days 1 (POD1), 3 (POD3), and 7 (POD7). Additionally, propranolol mitigated the beneficial effects on regulatory T cell (Treg) responses, diminishing the elevation of Tregs induced by surgical stress in breast cancer patients undergoing radical mastectomy (34). Propranolol can delay tumor progression in the MCA205 fibrosarcoma model and the MC38 colon cancer model, increase T cell infiltration, reduce MDSC infiltration, improve the survival rate of tumor-bearing mice, and TAMs to upregulate PD-L1 while altering their chemokine expression profile. This combination may also enhance the antitumor efficacy of ICIs (35, 36). In a mouse model of pancreatic cancer, ICIs alone, atenolol combined with ICIs, or highly selective β2 receptor blockers (ICI118551) combined with ICIs were ineffective in shrinking tumors; however, propranolol—a non-selective β-receptor antagonist—was able to sensitize tumors that were otherwise resistant to immunotherapy. Additionally, markers of T-cell exhaustion, such as PD-1 and TIM-3, exhibited increased expression in these tumors (36).

#### 2.2.2 α2 receptor agonists

α2 receptor agonists can directly activate macrophages and promote the recruitment and activation of CD4+ and CD8+ T lymphocytes within the tumor micro-environment. When administered alone, α2 agonists have demonstrated potent antitumor effects across various immunocompetent tumor models, primarily by targeting host cells rather than tumor cells (37).

#### 2.2.3 AT1 receptor antagonist

The AT1 receptor antagonist losartan has been shown to reverse the tumor micro-environment in pancreatic cancer and improve overall survival rates among patients. Losartan can downregulate the expression of immunosuppressive and invasion-related genes in PDAC. Results from phase II clinical trials indicated that, when combined with chemoradiotherapy, losartan significantly reduced the number of immunosuppressive regulatory T cells and FOXP3+ tumor cells within PDAC lesions, increased the infiltration of anti-tumor CD8+ T cells, and readjusted the immunosuppressive micro-environment (38).

However, not all anti-hypertensive agents demonstrate positive antitumor effects; for instance, spironolactone and furosemide attenuate the tumor-suppressive efficacy of PD-1 antibodies, while losartan and hydrochlorothiazide promote tumor growth and diminish the effectiveness of immunotherapy (39). Conversely, verapamil inhibits tumor growth by modulating NFAT2 expression and enhancing the immune response to PD-1 antibodies, making it a viable treatment option for cervical cancer, particularly in patients with hypertension (40). In summary, antihypertensive drugs present new application prospects in regulating the tumor immune micro-environment and augmenting the effects of immunotherapy. Nevertheless, further clinical studies are required to validate the safety and efficacy of these findings to determine optimal medication strategies that maximize their potential in tumor immunotherapy.

### 2.3 The sensitizing effects of weight-loss drugs on tumor immunotherapy

In recent years, research on weight-loss medications in the medical field has expanded beyond traditional approaches focused on calorie control and weight management, increasingly highlighting their potential value in tumor immunotherapy. These weight-loss agents primarily facilitate weight reduction by influencing fat absorption, suppressing appetite, enhancing satiety, and improving energy expenditure (41). Although there are currently no specific weight-loss drugs available in the domestic market, existing agents such as orlistat (a fatty acid synthase (FASN) inhibitor) and GLP-1 inhibitors have demonstrated significant weight loss effects.

More importantly, studies have shown that weight-loss medications possess considerable application prospects in tumor immunotherapy. They primarily function through two mechanisms: first, by improving the TME, which enhances the activity and functionality of immune cells by reducing the accumulation of inflammatory factors and cells; second, by augmenting the anti-tumor immune response and activating the immune system to release cytokines such as IFN-γ and IL-2, thereby inhibiting tumor growth and metastasis.

Fatty acid binding protein 1 (FABP1) is overexpressed in various tumors and is closely associated with tumor progression. FASN inhibitors, such as orlistat, can significantly inhibit FABP1 activity. The combined use of these inhibitors with PD-1 inhibitors has demonstrated a synergistic anti-tumor effect in cases of hepatocellular carcinoma (HCC) (42). Additionally, FASN inhibitors promote the infiltration and activation of CD8+ T cells by upregulating the expression of MHC-I, thereby enhancing the efficacy of tumor immunotherapy. It has also been shown that the combination of two distinct FASN inhibitors, orlistat and TVB-2640, with anti-PD-L1 antibodies significantly inhibits tumor growth *in vivo (* 43). Notably, FASN inhibitors markedly enhance the therapeutic effect of PD-1 monoclonal antibodies in patients with intrahepatic cholangiocarcinoma (ICC) associated with liver fluke infection (44), presenting a promising new treatment strategy for liver fluke-related ICC. Furthermore, GLP-1 inhibitors—widely utilized in recent years for their glucose-lowering and weight-loss effects—can restore the tumor-killing capacity NK cells (27).

In conclusion, weight-loss pharmacotherapies are increasingly recognized as promising adjuncts to cancer immunotherapy due to their ability to enhance the TME and bolster anti-tumor immune responses. Research on FASN inhibitors and GLP-1 analogues presents novel strategies for cancer immunotherapy that warrant further attention. Nevertheless, additional investigations and clinical trials are essential to assess potential adverse effects, evaluate efficacy across various tumor types, and determine the safety and effectiveness of combination therapies.

### 2.4 Antifungal drugs as tumor immunotherapy sensitizers

The role of antifungal agents in tumor immunotherapy represents a burgeoning area of research. These agents undergo metabolic processes within the body, inhibiting the activity of the CYP3A4 enzyme in the liver, which serves as both a common metabolic pathway for triazole antifungals and a crucial enzyme for the metabolism of immunosuppressive drugs. Antifungal agents have been shown to suppress tumor growth and proliferation, induce apoptosis in tumor cells, inhibit angiogenesis and various signaling pathways (45, 46), participate in modulating immune responses against tumors, and enhance DNA repair mechanisms along with overall anti-tumor efficacy (47). Investigating combinations of these agents with drugs targeting distinct pathways or cancer stem cells, as well as their potential roles in immune modulation, merits further exploration.

Recent *in vitro* studies have demonstrated that itraconazole can repolarize tumor-promoting M2 tumor-associated macrophages into an anti-tumor M1-like phenotype (48), which subsequently inhibits the proliferation of cervical cancer cells (49). While research has indicated the potential role of antifungal agents in tumor immunotherapy, further investigation is required to elucidate their specific mechanisms of action, efficacy, and safety. As research advances, antifungal drugs may emerge as significant adjuvant therapies for tumor immunotherapy.

### 2.5 Antiviral drugs as tumor immunotherapy sensitizers

In the realm of antiviral therapy, IFN-α serves as an endogenous regulator that has demonstrated significant antiviral activity at multiple critical stages of the viral replication cycle. Notably, its role in tumor immunotherapy is equally compelling. In this context, IFN-α not only directly activates innate and adaptive immune cells, including CD4+ T cells, CD8+ T cells, and NK cells, but also markedly enhances CTL-mediated cytotoxicity by promoting the expression of class I MHC molecules, thereby playing a crucial role in tumor immune editing processes. Subsequent studies have revealed that the combination of IFN-α with PD-1 blockade exhibits pronounced synergistic effects across various tumor models, including HCC.

*In vitro* studies have demonstrated that IFN-α increases tumor PD-L1 expression, while PD-1 blockade enhances IFN-α production, resulting in synergistic anti-tumor effects in a B16 melanoma mouse model (50). Clinical phase Ib/II trials have also validated the safety and efficacy of pembrolizumab in combination with PEG-IFN for patients with advanced melanoma (51). Furthermore, the combination of PEG-IFNα and PD-1 blockade significantly promotes T cell infiltration—particularly cytotoxic CD8+ T cells—into the tumor micro-environment, thereby improving the efficacy of PD-1 antibodies and prolonging survival in mice. In HCC, PEG-IFNα attracts CD8+ T cells by inducing CCL4 chemokine expression and upregulates PD-1 levels on these cells; subsequent PD-1 blockade restores T cell-mediated cytotoxicity, achieving a synergistic therapeutic effect against HCC (52).

It is noteworthy that the efficacy of IFN-α in postoperative adjuvant therapy for HCC has been clinically acknowledged and is recommended as one of the preferred treatment options by the latest clinical practice guidelines (53). Domestic studies have also elucidated a novel mechanism through which IFN-α can reshape the tumor immune micro-environment by modulating glucose metabolism in HCC cells, thereby enhancing immunotherapeutic effects. This discovery further substantiates the strategy of combining IFN-α with ICIs, such as PD-1 antibodies, for treating advanced HCC, demonstrating significant efficacy in tumor reduction, metastasis suppression, and survival prolongation (54).

In summary, IFN-α demonstrates significant potential across the domains of antiviral therapy, immune regulation, and tumor immunotherapy. Particularly in the treatment of HCC, the combinatorial strategy involving IFN-α offers a novel approach to immunotherapy, holds promise for addressing ICI treatment resistance, and represents an important clinical application for repurposing established drugs.

### 2.6 The role of mood stabilizers in enhancing tumor immunotherapy

Lithium carbonate (LC), recognized as a mood stabilizer, is extensively employed in clinical practice to treat psychiatric disorders such as mania, bipolar disorder, and depression by modulating the release and reuptake of neurotransmitters while promoting the synthesis of specific neurotransmitters like serotonin (55). However, in recent years, the potential role of lithium carbonate in tumor immunotherapy has garnered increasing attention.

Research has demonstrated that the accumulation of lactate within the TME is intricately linked to tumor progression and prognosis. Elevated levels of lactate can impair the functionality of TILs, rendering them immunosuppressive (56), which subsequently fosters tumor growth (57). LC inhibits lysosomal acidification by disrupting proton pump activity and activates specific signaling pathways that facilitate the transport of lactate from the cytoplasm into mitochondria, serving as an energy substrate for CD8+ T cells. This mechanism not only mitigates lactate-induced immunosuppression but also stimulates tumor-reactive CD8+ T cells, indicating potential anti-tumor efficacy. In preclinical studies, lithium carbonate administered either alone or in conjunction with PD-1 monoclonal antibodies has been shown to effectively inhibit tumor growth and extend the survival of mice. Furthermore, treatment with lithium carbonate enhances the activity of tumor-infiltrating CD8+ T cells in colon and breast cancer patients exhibiting elevated lactate levels, indicating the potential of targeting lactate metabolism as a novel strategy for anti-tumor therapy. Subsequent investigations revealed that lithium carbonate augmented the functional capacity of macrophages when delivered in nanoparticle form, enhancing their ability to produce nitric oxide, which may play a role in modulating immune responses within the TME. By significantly altering lysosomal pH and rescuing the diacylglycerol-protein kinase C (PKC) signaling pathway, lithium carbonate facilitates the localization of monocarboxylic acid transporter 1 (MCT1) (58) to the mitochondrial membrane and converts lactate into energy for CD8+ T cells, thereby counteracting the immunosuppressive effects induced by lactate. Additionally, alternative strategies have been proposed, including targeting MCT1 to diminish lactate uptake and inhibiting lactate dehydrogenase A (LDHA) to prevent lactate production, aimed at directly reducing lactate levels and activating T-cell-mediated anti-tumor immunity. In specific experiments, the administration of nano-lithium carbonate (nano-LC) particles into a male mouse model of liver cancer significantly enhanced macrophage functional activity, as evidenced by increased nitric oxide levels, further substantiating the beneficial role of lithium carbonate in modulating the tumor immune micro-environment (59). The assessment of macrophage functional activity following LC nanoparticle injection after liver cancer onset in male mice was conducted through measurements of nitric oxide production, arginase activity, and phagocytosis of zymosan particles. It was observed that nitric oxide levels produced by macrophages progressively increased, indicating that LC nanoparticles positively influence macrophage functionality during liver cancer progression (60).

In conclusion, lithium carbonate has great potential as a mood stabilizer in the field of tumor immunotherapy. As a strategy to target lactate metabolism, LC provides a new avenue for tumor immunotherapy. In the future, more clinical trials and research are needed to verify its efficacy and safety. We cannot ignore the potential side effects of LC; thus, continued study of its mechanism and safety is essential to better apply it in tumor immunotherapy.

### 2.7 The role of Disulfiram in enhancing tumor immunotherapy

Disulfiram (DSF), originally developed as an anti-alcohol addiction medication, is emerging as a potential non-chemotherapeutic adjuvant anticancer agent (61). Molecular encapsulation with cyclodextrin enhances the solubility and stability of DSF. The metal complexes of DSF exhibit tumor cell growth inhibition through various mechanisms. Further investigations have demonstrated that DSF can induce apoptosis in tumor cells by arresting the cell cycle, inhibiting mitochondrial function, and enhancing antitumor immunity (62). Beyond its direct cytotoxic effects, DSF effectively induces immunogenic cell death (ICD) (63) in cancer cells and modulates the immunosuppressive tumor micro-environment, thereby augmenting ICI therapy and triggering systemic immune responses (64–66).

DSF has demonstrated multiple mechanisms of action in anti-tumor immunotherapy, significantly enhancing its antitumor efficacy. Both animal and *in vitro* studies have indicated that DSF induces apoptosis in CRC cells while upregulating the expression of key signaling molecules associated with ICD, such as calreticulin and heat shock protein 70 (66), thereby activating antitumor immune responses. Furthermore, DSF enhances T cell functionality, interleukin-2 production, T cell proliferation, and metabolic reprogramming by directly binding to and activating lymphocyte-specific protein tyrosine kinase (LCK). Notably, the activation of CD8+ T cells exhibits potent antitumor immune effects against melanoma and colon cancer, which are further amplified when combined with PD-1 inhibitors (67).

Additionally, DSF inhibits tumor growth and metastasis by modulating the function of the chemokine signaling regulator FROUNT, thereby affecting the activity of tumor-associated macrophages (TAMs), reducing intratumoral macrophage accumulation, and inhibiting their functionality while simultaneously increasing the population of cytotoxic CD8+ T cells (68). DSF binds to copper (Cu²^+^) ions, inhibits glycogen synthase kinase 3 beta (GSK3β) activity through PARP1 signaling inhibition, upregulates programmed death-ligand 1 (PD-L1) expression, and synergistically enhances the efficacy of anti-PD-1 antibodies to effectively slow HCC progression (69). Furthermore, DSF/Cu complexes augment the effectiveness of CD47 blockade, promote dendritic cell maturation, and further enhance CD8+ T cell cytotoxicity via ICD-mediated immune activation (70).

The DSF/Cu complex also promotes type I interferon release and DC maturation, demonstrating enhanced efficacy in the treatment of metastatic breast cancer when combined with cytotoxic agents such as docetaxel (DTX), compared to monotherapy, thereby providing a novel therapeutic strategy for patients with metastasis (71). In non-small cell lung cancer (NSCLC), the combination of DSF with anti-PD-L1 effectively overcomes drug resistance and enhances antitumor effects by modulating hypoxia-inducible factor 1 (HIF-1) signaling (72). Additionally, DSF activates the STING signaling pathway through PARP1 inhibition and upregulates the expression of IFNα and IFNβ, which, in conjunction with chemoimmunotherapy, significantly inhibits PDAC tumor growth in murine models (73). Finally, DSF exhibits superior antitumor efficacy compared to monotherapy in triple-negative breast cancer (TNBC) by enhancing PD-L1 expression via DNMT1-mediated hypomethylation of IRF7 (74).

Taken together, DSF enhances anti-tumor immune responses through multiple mechanisms, providing new hope and directions for the treatment of a variety of cancers.

### 2.8 Analysis and prospects

The repurposing of established drugs in tumor immunotherapy holds significant importance, primarily reflected in three key aspects: accelerating the research and development process, shortening timelines, and reducing costs. Since these drugs have already undergone clinical validation, they can swiftly progress to trial phases with well-established pharmacological profiles and safety data, thereby mitigating research and development risks. Additionally, the identification of new indications allows for the exploration of novel therapeutic targets or pathways that can substantially enhance immunotherapeutic efficacy. For instance, hypoglycemic agents, antihypertensives, lipid-lowering medications, psychotropic drugs, and antibiotics have demonstrated potential anti-tumor effects. By broadening their applications, repurposed drugs can rapidly address clinical needs while expanding the therapeutic scope of marketed products and providing timely and effective treatment options for patients.

**Figure 3:**
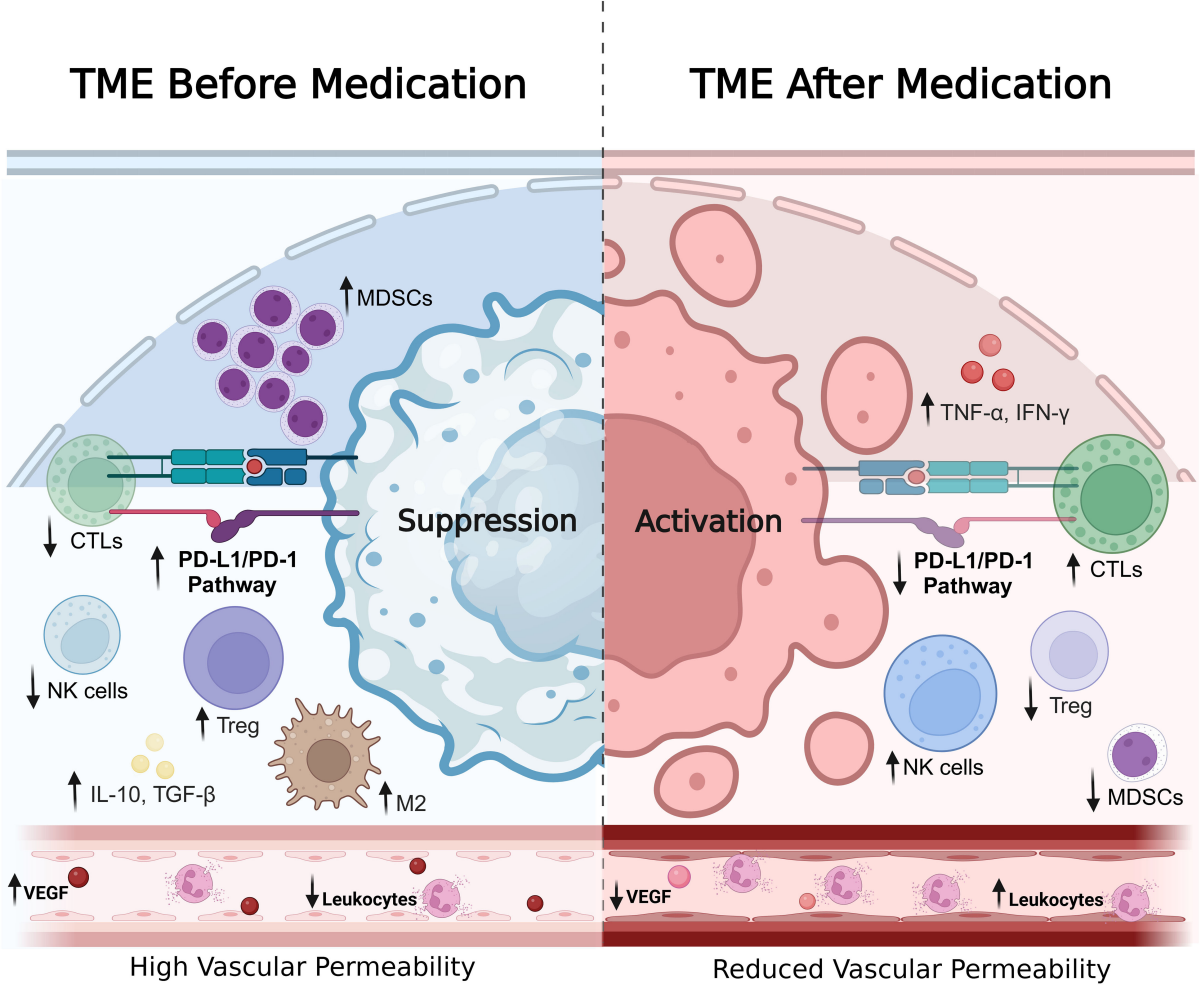
Changes in TME before and after drug treatment. This figure illustrates the alterations in the tumor microenvironment before and after drug intervention, emphasizing contrasts in cell composition, signaling pathways, vascular properties, and immune cell infiltration. The left panel depicts the tumor microenvironment prior to treatment, characterized by a substantial presence of myeloid-derived suppressor cells MDSCs, M2 macrophages (M2), and regulatory Tregs, all contributing to an immunosuppressive state. The PD-L1/PD-1 pathway is highly active, facilitating tumor cell evasion of immune surveillance. Vascular structures exhibit increased permeability due to elevated levels of angiogenic factor vascular endothelial growth factor (VEGF), which supports tumor proliferation and metastasis. Concurrently, leukocyte infiltration is limited at this stage; thus, the capacity for immune cells to penetrate the tumor microenvironment is compromised, further exacerbating immunosuppression while high concentrations of immunosuppressive cytokines such as IL-10 and TGF-β prevail. The right panel presents the tumor microenvironment following drug treatment, where there is a marked increase in activation of CTLs and natural NK cells, accompanied by a significant reduction in Treg and MDSC populations. Inhibition of the PD-L1/PD-1 pathway renders the immune system more active while effectively diminishing tumor cell immune evasion capabilities. At this juncture, features indicative of apoptosis are observed within tumor cells—signifying a pronounced effect from enhanced immune responses on these malignant entities. Vascular permeability decreases due to reduced VEGF levels, thereby limiting nutrient access for tumors and impairing their metastatic potential; additionally, white blood cell infiltration increases significantly allowing more immune cells to effectively enter the tumor microenvironment for targeted attack against cancerous tissues. Levels of pro-inflammatory cytokines such as TNF-α and IFN-γ rise substantially further amplifying anti-tumor immunity. The contents depicted in this figure underscore the dynamic influence of drug therapy on the TME, highlighting its transition from an immunosuppressive state to one characterized by immune activation—a critical aspect for effective cancer immunotherapy.

The combination of established drugs with immunotherapeutic agents has emerged as a promising strategy to significantly enhance tumor therapeutic efficacy. This approach primarily works through several mechanisms: improving the tumor micro-environment by reducing inflammation and immunosuppression, enhancing immune cell function, increasing the sensitivity of tumor cells to immune recognition, and regulating other immune system components that affect the recruitment and activation of immune cells. This comprehensive approach effectively reduces immune evasion and inhibits tumor cells from escaping immune attack (**Figure 3**).

In exploring the indications for established drugs in immunotherapy, it is crucial to consider not only their direct antitumor effects but also their potential interactions with various immune pathways. One such important axis is the PD-1/PD-L1 pathway, which has demonstrated significant efficacy in various tumor immunotherapies. However, it is important to recognize that the PD-1/PD-L1 axis is not the sole mechanism by which tumors evade immune surveillance and clearance. For instance, during T-cell activation, there is a complex interplay between the CTLA-4 and PD-1 pathways. Anti-CTLA-4 treatment can increase PD-1 expression, while anti-PD-1 treatment can reverse CTLA-4-mediated T-cell dysfunction. Similarly, the IDO and TGF-β pathways also exhibit complex interactions with the PD-1/PD-L1 axis, jointly regulating tumor immune escape. These interactions underscore the need for a more holistic approach to tumor immunotherapy that targets multiple pathways simultaneously. Furthermore, in combination tumor therapies, older drugs can also play a pivotal role in enhancing tumor immune responses. By activating AMPK, inhibiting the IL-6/JAK2/STAT3 pathway, and regulating metabolic pathways, these drugs can further bolster the antitumor efficacy of immunotherapies. Given the intertwined nature of these immunosuppressive mechanisms within the tumor microenvironment, the combined use of older drugs with immunotherapies targeting these pathways may produce synergistic effects, leading to even better clinical outcomes. illustrates the multifaceted mechanisms by which established drugs and immunotherapies can work together to enhance tumor therapeutic efficacy.

The analgesic and anesthetic agent ketamine has been shown to participate in immunomodulation by inhibiting mRNA synthesis in lipopolysaccharide (LPS)-activated macrophages, thereby reducing the production of TNF-α, interleukins, and nitric oxide, which subsequently affects macrophage-mediated immunity. The role of ketamine in antitumor therapy remains unexplored (75). Pitavastatin effectively inhibits the expression of IL-33 and prevents the progression from chronic inflammation to tumors by blocking activation of the TBK1-IRF3 signaling pathway (76), which triggers Th2 cell activation and type 2 innate lymphocyte (ILC2) responses that drive type 2 immune reactions. Hydroxychloroquine is primarily utilized for rheumatic diseases, cardiovascular conditions, and chronic kidney disorders; it also exhibits immunomodulatory effects and anti-inflammatory properties. However, there is currently no relevant research within the realm of tumor immunity (77, 78), which opens up new potential for hydroxychloroquine as a multi-system anticancer agent with high efficacy and low toxicity (79), warranting further investigation in the field of tumor immunotherapy. In recent years, numerous in-depth studies have demonstrated that Traditional Chinese Medicine (TCM) exhibits significant potential in regulating the TME, not only reversing the resistance to ICI but also enhancing clinical treatment outcomes and effectively mitigating the severity of immune-related adverse events (80). Specifically, in various tumor-bearing mouse models, artemisinin has been found to inhibit the functional activity of MDSCs in the TME by inducing the transition of macrophages from the M2 to M1 phenotype (81). Furthermore, in lung cancer models, the active ingredient cryptotanshinone from Salviae Miltiorrhizae Radix has been shown to increase the infiltration levels of CD8+ T cells and CD4+ T cells in the TME (82). Similarly, in breast cancer animal models, another extract from Salviae Miltiorrhizae Radix, Salviaric acid B, has demonstrated the ability to enhance the infiltration of CD8+ T cells in the TME (83), thereby amplifying the antitumor effect of PD-1 blockade therapy. Additionally, in experiments targeting the mouse melanoma B16 model, intranasal administration of astragalus polysaccharides activated DC in mesenteric lymph nodes and promoted the activity of NK and T cells, significantly enhancing the antitumor action of anti-PD-L1 monoclonal antibody (mAb) (84).

The clinical efficacy of many repurposed drugs in cancer treatment has thus far been demonstrated only in a subset of patients (85), highlighting the urgent need for systematic workflows and simplified strategies to identify potentially repurposed drugs for enhancing ICI therapy in the era of personalized medicine. To address this challenge, several systematic approaches have been developed for identifying drug repurposing candidates in tumor immunotherapy. These include high-throughput screening (HTS) of existing drug libraries against tumor-immune interaction models to identify immunomodulatory agents (86, 87), computational repositioning using machine learning algorithms to analyze drug-target interactions and predict potential candidates (88), bioinformatic integration of multi-omics data to pinpoint drugs that reverse immune suppression or enhance immune activation (89), CRISPR/Cas9 functional screening to map genome-wide knockout targets to approved drugs (90, 91), and network pharmacology to prioritize candidates with polypharmacological effects on immune checkpoints or tumor microenvironment modulation (92).

To further support drug repositioning studies in immunotherapy, several publicly accessible databases can also be leveraged. These include DrugBank (https://go.drugbank.com), a comprehensive repository of FDA-approved drugs with detailed annotations on targets, pathways, and clinical trial data; ImmPort (https://www.immport.org), which shares datasets from immunotherapy studies, including immune cell profiles and cytokine responses; RepoDB (http://apps.chiragjpgroup.org/repoDB), curating successes and failures of drug repositioning across diseases; ClinicalTrials.gov (https://clinicaltrials.gov), tracking ongoing trials of repurposed drugs in cancer immunotherapy; and PharmGKB (https://www.pharmgkb.org), linking drug-gene interactions to immune-related pathways to aid in mechanistic hypothesis generation (93). Collectively, these databases provide valuable resources for identifying and validating drug repurposing candidates in the context of tumor immunotherapy.

The exploration of established drugs in tumor immunotherapy encompasses several directions: modifying drug delivery methods to enhance efficacy and safety, investigating new therapeutic applications, optimizing dosage and administration, and integrating with novel therapies. Drug function retargeting holds significant promise in anti-tumor immunotherapy. It is essential to conduct comprehensive mechanistic studies, integrate treatment approaches, evaluate safety profiles, and foster interdisciplinary collaboration to provide more effective treatment options for cancer patients.
